# Analgesic and Anti-inflammatory Effects of *Rosa damascena* Hydroalcoholic Extract and its Essential Oil in Animal Models

**Published:** 2010

**Authors:** Valiollah Hajhashemi, Alireza Ghannadi, Mohammad Hajiloo

**Affiliations:** a *Department of Pharmacology and Isfahan Pharmaceutical Sciences Research Center, School of Pharmacy and Pharmaceutical Sciences, Isfahan University of Medical Sciences, Isfahan, Iran.*; b ^b^*Department of Pharmacognosy, School of Pharmacy and Pharmaceutical Sciences, Isfahan University of Medical Sciences, Isfahan, Iran.*

**Keywords:** *Rosa damascene*, Rosaceae, Analgesic, Anti-inflammatory

## Abstract

Extracts obtained from the petals of *Rosa damascena *(Rosaceae) are used in Iranian folk medicine as remedies for the treatment of some inflammatory diseases. In this study the hydroalcoholic extract and essential oil of the plant were investigated for its possible anti-inflammatory and analgesic activities. The extract was administered at the doses (p.o.) of 250, 500 and 1000 mg/kg and the doses of essential oil were 100, 200 and 400 μL/kg. The acetic acid-induced writhing response, formalin-induced paw licking time in the early and late phases and light tail flick test were used in mice to assess analgesic activity. For evaluation of anti-inflammatory effect carrageenan-induced paw edema served as a valid animal model in rats. The extract significantly attenuated the writhing responses induced by an intraperitoneal injection of acetic acid and also showed potent analgesic effect in both phases of formalin test but not in light tail flick test. In addition, the higher dose of the extract significantly (P < 0.05) reduced carrageenan-induced paw edema. Essential oil of the plant at all administered doses failed to show any analgesic or anti-inflammatory effect in above mentioned tests. These results provide support for the use of hydroalcoholic extract of *Rosa damascena *in relieving inflammatory pain, and insight into the development of new agents for treating inflammatory diseases.

## Introduction

Inflammation is a pathophysiological response of mammalian tissues to a variety of hostile agents including infectious organisms, toxic chemical substances, physical injury or tumor growth leading to local accumulation of plasma fluid and blood cells (1). The non-steroidal anti-inflammatory drugs (NSAIDs) such as acetyl salicylic acid (aspirin), diclofenac sodium, ibuprofen and their new congeners, namely selective COX-2 inhibitors such as celecoxib exert their anti-inflammatory effects via inhibition of early steps in the biosynthesis pathway of prostaglandins and are widely used for managing inflammation and pain (2, 3). However, the side effects of the currently available anti-inflammatory drugs including gastric injury and ulceration, renal damage, and bronchospasm (4) and cardiac abnormalities especially for newer drugs such as rofecoxib and celecoxib (5) has limited their use. Corticosteroids also have potent anti-inflammatory activity but again their multiple adverse effects limit their uses (3). Therefore, a need arises for the development of newer anti-inflammatory agents probably from the natural origin with more powerful activity and with lesser side effects to substitute the current chemical therapy. 

*Rosa damascena *or Persian rose is a small plant belonging to the Rosaceae family. It is a small plant with aromatic light pink flowers, which appear in spring (6) and today are highly cultivated all over the world, including Iran (especialy in Kashan), Turkey, India, Bulgaria for visual beauty and its use in production of fragrances (7, 8). This plant contains flavonoids such as caempferol and quercetin and their glycoside derivatives (9,10), carboxylic acids (11) terpene, myrcene, tannins and vitamin C (8). Essential oil obtained from *Rosa damascena *cultivated in central Iran is mainly consisted of beta-citronellol, nonadecane, geraniol and docosane (12). In addition to its perfuming effect, flowers, petals and hips (seed-pot) of *Rosa damascena *are used for medical purposes. It has been used as cardiotonic (13), mild laxative (14), anti-inflammatory (11), cough suppressant (8) and also for the treatment of menstrual bleeding and digestive problems (15).

Recent studies demonstrated anti-HIV (9), anticonflict (16), antibacterial (17), antitussive (18) and respiratory smooth muscle relaxant (19) properties for this plant. Anti-inflammatory and antinociceptive activities have been reported for another species of rosa genus, namely *Rosa hybrida *(20). Based on traditional uses of the plant in inflammatory conditions and also anti-inflammatory and antinociceptive effects reported for another species of Rosa genus, this study aimed to find out pharmacological evidences for analgesic and anti-inflammatory effects of *Rosa damascena *using standard animal models.

## Experimental

*Plant material and preparation of extract and essential oil*

The petals of the plant were purchased from a local market and the scientific name of the plant was confirmed by Department of Botany (School of Sciences, Isfahan University, Isfahan, Iran). A voucher specimen (No. RD-112) was deposited in the Herbarium of Faculty of Pharmacy and Pharmaceutical Sciences, Isfahan University of Medical Sciences, Isfahan, Iran.

For preparation of hydroalcoholic extract, air-dried and powdered petals of the plant (200 g) were macerated with 1500 mL of EtOH-H_2_O (7:3) for 48 h. The extract was then shaked, filtered and evaporated in a rotating evaporator under reduced pressure until dryness (21). 

The essential oil was isolated by hydrodistillation of the air-dried powdered petals of the plant for 3 h according to the method recommended in European Pharmacopoeia (22). 

*Animal models and habituation*

Male wistar rats and male mice, weighing 160-200 and 25–35 g, respectively, were used in this study. Animals were housed in groups of six per standard makrolon cage, on 12-h light/12-h dark cycle; and air temperature was maintained at 22 ± 2°C. They were offered food and water *ad libitum*. Experiments reported in this study were carried out in accordance with local guidelines for the care of laboratory animals of Isfahan University of Medical Sciences. 

*Writhing test*

This test is performed in mice according to the method described by Ferreira et al. (23) with slight modifications. 1% acetic acid solution (10 mL/kg, b.w.) was injected intraperitoneally. Animals were pretreated with *R. damascena *extract (250, 500 and 1000 mg/kg), essential oil (100, 200 and 400 μL/kg) or indomethacin (10 mg/kg) orally 45 min. prior to the peritoneal irritation. Control animals received the same volume of 0.9% NaCl solution. The resulting writhes and stretching were observed and counted over a period of 10 min. starting 10 min. after acetic acid injection.

*Formalin test*

The method used in the present study was adapted from de Miranda et al. (24) with slight modifications. It consists briefly of injecting subcutaneously 20 microliter of 2.5% formalin into the right posterior paw of mice placed in a transparent enclosure. Throughout 5 min prior to this procedure, each mouse is allowed to adapt the testing box and left freely moving and exploring (habituation). The formalin-induced licking of the paw was considered as indicative of the nociceptive behaviour. Using a chronometer, the total time spent in licking and biting the injected paw is recorded 0-5 and 20-30 min. after formalin injection.

In this test, hydroalcoholic extract (250, 500 and 1000 mg/kg) and essential oil (100, 200 and 400 μL/kg) of *R. damascena *were administered orally 45 min. prior to formalin injection. Control group received isotonic saline 0.9% (10 mL/kg) and a group of animals received morphine (10 mg/kg, i.p.) as a standard analgesic drug.

*Light tail flick test*

Acute nociception was assessed using a tail flick apparatus (Pooya-armaghan, Iran) according to the method of D^,^Amour and Smith (25). Briefly, each animal was placed in a restrainer, 2 min before treatment, and baseline reaction time was measured by focusing a beam of light on the distal one-third portion of the animals tail. The same doses of extract, essential oil, morphine and vehicle used in formalin test, were administered orally and 30 min. later the post drug reaction time was measured at 15 min intervals until 2 h. A 12 sec cut-off time was used in order to prevent tissue damage. The MPE% (percent of maximum possible analgesic effect) was calculated for each time interval. Doses of the extract, essential oil and morphine were the same as in formalin test.

*Anti-inflammatory activity*

The anti-inflammatory activity was evaluated by the carrageenan-induced paw edema test in the rat (26). Male wistar rats (160-200 g) were briefly anaesthetized with ether and injected subplantarly into right hind paw with 0.1 mL of 1% suspension of carrageenan in isotonic saline. The left hind paw was injected with 0.1 mL saline and used as a control. Paw volume was measured prior and 4 h after carrageenan administration using a mercury plethysmorgraph (Ugo Basil, Italy). 

*R. damascena *extract and essential oil were administered 1 h prior to carrageenan injection. The control group received equivalent volume of the vehicle. Indomethacin (10 mg/kg) was used as positive control. 

*Data analysis*

Data obtained were expressed as mean ±SEM. Differences between groups were statistically analyzed by one-way analysis of variance (ANOVA) followed by Duncan as the post hoc test. Significance was defined at P < 0.05 level. 

## Results

*Pharmacognosy*

Evaporation and solvent removal of hydroalcoholic extract gave a semi-solid mass with a yield of 37.5% and the yield of essential oil was 0.025 % (v/w).

*Pharmacology*

In acetic acid-induced writhing, essential oil of *R. damascena *failed to show any analgesic effect, while hydroalcoholic extract at doses of 500 and 1000 mg/kg significantly (P < 0.001) inhibited abdominal twitches (Table 1).

**Table 1:** Preventive effect of *Rosa damascena* hydroalcoholic extract and its essential oil on acetic acid-induced writhing in mice (n = 6).

**Treatment**	**Dose**	**Number of writhes (mean ± SEM)**	**Percent inhibition**
Control	-	51.8 ± 4.3	-
HE	250 (mg/kg)	43.8 ± 5.2	15
	500 (mg/kg)	31.2 ± 4.8*	40
	1000 (mg/kg)	26.8 ± 3.3*	48
EO	100 ( μl/kg)	49.1 ± 1.5	5
	200 ( μl/kg)	47.3 ± 2.8	9
	400 ( μl/kg)	45.8 ± 4.0	12
Indomethacin	10 (mg/kg)	12.1 ± 4.4*	78

* P < 0.05 compared with control group. HE: Hydroalcoholic extract; EO: Essential oil.

In formalin test again essential oil of the plant at doses of 100, 200 and 400 μL/kg could not reduce time spent for paw licking but hydroalcoholic extract reduced paw licking time of both phases of formalin test in a dose dependent manner (Table 2). In light tail flick test, while morphine as a reference drug produced a potent analgesia, both essential oil and hydroalcoholic extract were ineffective (data not shown). 

**Table 2:** Preventive effect of *Rosa damascena* hydroalcoholic extract and its essential oil in the formalin test (n = 6).

**Treatment**	**Dose**	**Paw licking time (sec)**			
		**First phase (0-5 min)** **(mean ± SEM)**	**Inhibition (%)**	**Second phase (20-30 min) ** **(mean ± SEM)**	**Inhibition (%)**
Control	-	68.1 ± 8.7		109.2 ± 12.2	-
HE	250 (mg/kg)	48.5 ± 2.7	29	64.0 ± 6.1	41
	500 (mg/kg)	38.8 ± 5.7a	43	51.2 ± 8.2a	53
	1000 (mg/kg)	27.6 ± 3.3b	59	38.5 ± 9.8 b	65
EO	100 ( μl/kg)	62.2 ± 7.6	9	93.0± 8.6	15
	200 ( μl/kg)	43.6 ± 5.3	36	76.7 ± 6.1	30
	400 ( μl/kg)	40.6 ± 8.7	40	69.4 ± 6.9	36
Morphine	10 (mg/kg)	5.0 ± 0.6b	93	3.3 ± 0.4b	97

*a* P < 0.01 and *b*P < 0.001 compared with control group.

HE: Hydroalcoholic extract; EO: Essential oil.

The results of carrageenan test have been summarized in Table 3. In this test, the hydroalcoholic extract of the plant only at a dose of 1000 mg/kg significantly (P < 0.05) reduced carrageenan-induced paw edema and essential oil at all administered doses had no anti-inflammatory activity. Indomethacin also significantly (P < 0.05) reduced inflammation so that edema was 76% less than control group. 

**Table 3:** Preventive effect of *Rosa damascena* hydroalcoholic extract and its essential oil against carrageenan-induced rat paw edema

**Treatment**	**Dose**	**Percent inhibition of paw edema**
Control	-	-
HE	250 (mg/kg)	11
	500 (mg/kg)	41
	1000 (mg/kg)	33*
EO	100 (μL/kg)	2
	200 (μL/kg)	0
	400 (μL/kg)	-3
Indomethacin	10 (mg/kg)	76*

*P < 0.05 compared with control group.

HE: Hydroalcoholic extract; EO: Essential oil.

## Discussion

The results obtained in this study lead us to confirm that *R. damascena *hydroalcoholic extract possesses a significant effect against pain in two frequently used antinociceptive models in mice. 

In acetic-acid induced writhing the hydroalcoholic extract in a dose dependent manner inhibited the abdominal constrictions. Although the pain in the abdominal writhes induced by acetic acid is not a specific model, the involuntarily muscle twitches of the abdomen may be of interest because of their similarity with some of those known in visceral disorders (27, 28). 

In the formalin test which is sensitive for various classes of analgesic drugs (29), our results showed that the time spent in licking the injured paw was significantly reduced by oral administration of the hydroalcoholic extract in both phases. In this test, the centrally acting drugs such as narcotics inhibited both phases equally, while peripherally acting drugs only inhibited the second phase (28, 29). It is also well known that the formalin model may involve sensorial C-fibers (30) in early phase and a combined process generated by peripheral inflammatory tissue and functional changes in the dorsal horn in late phase (31, 32). In fact, the effect of *R. damascena *extract on both phases showed that they contain active analgesic principles acting both centrally and peripherally. Recently it has been reported that antioxidants reduce pain of tonic (second) phase of formalin test (33). Petals of *R. damascena *contain several flavonoids (9, 10) and it has been reported that flavonoids have antioxidant properties (34). Therefore it seems that these compounds have some role in the analgesic effect. 

In light tail flick test, a central model which has a selectivity for opioid-derived analgesics (28) essential oil and hydroalcoholic extract could not exert any antinociceptive activity and it means that opioid receptors are not involved in analgesia which was observed in formalin and acetic acid-induced writhing and further studies are needed to find out the exact mechanism. 

It has been documented that carrageenan-induced rat paw edema is a suitable *in vivo *model to predict the value of anti-inflammatory agents, which act by inhibiting the mediators of acute inflammation (35). The method was chosen for this study since edema induced by carrageenan is the most prominent acute experimental model in search for new anti-inflammatory drugs (36). In addition, it is a method that has been frequently used to assess the anti-inflammatory effect of natural products (37, 38). In this test essential oil of *R. damascena *had no anti-inflammatory activity while the extract at a dose of 1000 mg/kg significantly (P < 0.05) reduced carrageenan-induced edema.

It is known that carrageenan-induced paw edema involves many mediators including histamine, serotonin, bradykinin (39, 40) and prostaglandins (41). Our work presents a primary study and further investigations are required to clarify the effect of the plant components on these mediators.

In conclusion, our results clearly demonstrated that hydroalcoholic extract of *R. damascena *has a potent analgesic effect in acetic acid and formalin tests and also showed anti-inflammatory activity in carrageenan model and these results provided enough credit for the plant use as a remedy against painful and inflammatory conditions.
